# Alteration in Inflammation-related miR-146a Expression in NF-KB Signaling Pathway in Diabetic Rat Hippocampus

**DOI:** 10.15171/apb.2016.015

**Published:** 2016-03-17

**Authors:** Fatemeh Habibi, Farhad Ghadiri Soufi, Rafighe Ghiasi, Amir Mahdi Khamaneh, Mohammad Reza Alipour

**Affiliations:** ^1^ Neurosciences Research Center, Tabriz University of Medical Sciences, Tabriz, Iran.; ^2^ Molecular Medicine Research Center, Hormozgan University of Medical Sciences, Bandar Abbas, Iran.; ^3^ Department of Physiology, Tabriz University of Medical Sciences, Tabriz, Iran.; ^4^ School of Advanced Medical Sciences, Tabriz University of Medical Sciences, Tabriz, Iran.

**Keywords:** Diabetes, Hippocampus, TRAF6, IRAK1, NF-KB, miR-146a

## Abstract

***Purpose:*** The purpose of the present study is to evaluate the expression of miR-146a gene, its adaptor genes (TRAF6, NF-KB, and IRAK1), and possible changes in the cellular signaling pathway in diabetic hippocampus tissue.

***Methods:*** Male Sprague–Dawley rats are randomly selected and divided into control and diabetic (n=6) groups. Diabetes induced by the single-dose injection of nicotinamide [110 mg/kg, (i.p.)], 15 min before streptozotocin (50 mg/kg; i.p.) in 12-h fasted rats. The rats are kept at the laboratory for two months. After anaesthetization, hippocampus of the rats was removed in order to measure the expression of miR-146a, NFK-B, IRAK1, and TRAF6 genes using real-time PCR and activity of NF-KB as well as amount of apoptosis rate using ELISA.

***Results:*** The results indicated a reduction in expression of miR-146a and an increase in expression of IRAK1, NF-KB, and TRAF6 genes in the hippocampus of diabetic rats compared to control. Also it reveals an increase in the activity of NF-KB and apoptosis rate in the hippocampus of diabetic rats.

***Conclusion:*** Our results report the probability that reduction of miR-146a expression in the negative feedback loop between miR-146a and NF-KB increases NF-kB expression and thus intensifies inflammation and apoptosis in hippocampus.

## Introduction

Diabetes mellitus is a complex disease which is characterized by chronic hyperglycemia with a disturbance in the metabolism of carbohydrates, fats, and proteins.^1^ This disease occurs due to the disturbance in insulin secretion, insulin operation, or both, and about 8% of the American population is suffering from it.^2-5^ In addition to the factors like food diet, lifestyle, and obesity, factors such as genetic history and changes in the gene expression have also been considered the effective factors for the development and progression of this disease.

Micro-ribonucleic acids (miRNAs), which play an important role in biologic and physiologic processes like proliferation, evolution, and differentiation and are considered one of the genes involved in diabetes, are a group of tiny, single-string genes (containing 20-32 nucleotides) that were discovered at the beginning of 1990s.^6^ These genes play a regulatory role in post-transcription and protein synthesis processes.^7^ Studies have shown that microRNA-146a (miR-146a) has a role in developing a disturbance in insulin secretion due to proinflammatory cytokines.^8^ Also, it has been reported that increase in miR-146a induction increases the programmed death of beta cells.^7^ Nuclear factor kappa B (NF-KB) is a transcription factor that is involved in the cellular response to the stimulations like proinflammatory cytokines such as tumor necrosis factor alpha (TNF-α) and interleukins (IL).^9^ Activation of NF-KB is an important event in the gradual reduction of beta cells during diabetes where preventing this process provides a protection for beta cells against apoptosis inducted by cytokine.^10^ Several studies have reported that diabetes, as an inflammatory disease, increases the level of NF-KB in most of the tissues in the body.^7-11^ Some studies have also indicated the appearance of inflammation in hippocampus among diabetic rats.^12^ Hippocampus is an important part of the brain for memory performance and plays an important role in cognitive and emotional behaviors.^13^ There are some reports about disturbance in memory, learning, and risk of dementia and stroke^14^ as well as cognitive damage from the synaptic plasticity in hippocampus among diabetics.^15^ Two key molecules in NF-KB pathway include kinase activating interleukin-1 receptor (IRAK1) and tumor necrosis factor receptor-associated factor (TRAF6) that are targeted to negative feedback regulation by miR-146a.^16^ It has been shown that increase in the expression of miR-146a can inhibit IRAK1 and TRAF6 expressions and then reduce NF-KB activity.^17^

As mentioned earlier, after the development of diabetes, its destructive effects are gradually observed in different organs of the body; and in most cases, inflammation has an important role in the manifestation of these effects. Furthermore, hippocampus has been proposed as one of the tissues whose operation can be hindered in this regard. Therefore, in this study, we measured the amount of expression of NF-KB, miR-146a, TRAF6 and IRAK1 genes as well as NF-KB activity and apoptosis rate in the hippocampus of diabetic rats.

## Materials and Methods

### Experimental design

Male Sprague–Dawley rats (Razi Institute, Tehran, Iran) weighing 300–330 g were housed at room temperature (22–25 °C) with 12:12 h light/dark cycles and free access to food and water. The study protocol was designed in accordance with the NIH guidelines for the care and use of animals and approved by the Ethics Committee for the Use of Animals in Research at Tabriz University of Medical Sciences (No: 91/2-2/5/4 Dec 2012). The rats are randomly divided into diabetic and control groups (n=6).

Diabetes was induced in the diabetic group by single-dose injection of nicotinamide [110 mg/kg; intraperitoneal (i.p.)], 15 min before injection of streptozotocin (STZ; 50 mg/kg dissolved in 0.1 M of citrate buffer; pH 4.5; i.p.) in 12-h fasted rats. Then, Forty eight hours after the injection of STZ, blood sugar over 250 mg/dl is considered a diabetic indicator. Control rats received an injection of citrate buffer alone. Two months after the diabetes induction, the rats in the both groups are anesthetized by the intraperitoneal injection of 80 mg/kg ketamine and blood samples (5 ml per rat) were collected from the heart. After cervical decapitation, hippocampus was quickly removed and frozen in liquid nitrogen.

### ELISA measurements

The plasma insulin concentration was determined by rat ELISA kit (Cayman chem., Ann Arbor, MI, USA; Cat. No: 589501). Also, the NF-kB activity in hippocampus was determined by measuring the phosphorylated NF-kB p65 levels with an ELISA kit (Cayman Chemical, Ann Arbor, MI), according to the manufacturer’s instructions. we measured apoptosis rate in this tissue using cell death detection ELISA kit (1544675, Roche, Germany) at 405 nm as previously described.^18^ According to the manufacturer’s instructions, 20-mg sections from hippocampus were homogenized in 400 μl of hypotonic buffer for 15 min and centrifuged at 14,000×g for 10 min at 4 °C. The supernatant was used for determination apoptosis rate. The remaining nuclear pellet was resuspended in 100 μl of extract buffer for 10 min and centrifuged at 14,000×g for 10 min at 4 °C. The supernatant containing the nuclear fraction was used for quantification of NF-kB activity.

### Real-time PCR

In this study mRNA and miRNA expressions in hippocampus tissue performed by real-time PCR as previously described.^19^ Total RNA was extracted from the hippocampus using the miRCURY RNA Isolation Kit (Exiqon, Vedbaek, Denmark) and RNA content were measured using Nanodrop 1000 spectrophotometer (Thermo scientific,Wilmington, DE, USA). The cDNA synthesis kit (Fermentas GmBH, Leon-Rot, Germany) were used for determination expression of miR-146a, IRAK1, TRAF6 and NF-kB genes. Each cDNA was used as a template for real-time PCR assay using the SYBR Green master mix (Exiqon, Vedbaek, Denmark). The locked nucleic acid (LNA) forward and reverse primer sets (Exiqon, Vedbaek, Denmark) for microRNA and mRNA have been listed in Table 1. Real-time PCRs were performed on a Bio-Rad iQ5 Detection System (Bio-Rad, Richmond, CA, USA). The relative amount of mRNA and miRNA for each target gene was calculated based on its threshold cycle (Ct) compared to the Ct of the housekeeping (reference) gene (β-actin and miR-191). The relative quantification was performed by 2^-ΔΔ Ct^ method.

The specificity of the PCR reactions was verified by generation of a melting curve analysis.

### Data analysis

Results are expressed as mean ± SD. Statistical analysis was performed using SPSS software (SPSS, Chicago, IL, USA; version 18). The Student's sample t-test used to compare variables between groups. A level of p<0.05 was considered statistically significant.

**Table 1:** Primer set list for mRNAs and miRNAs.

**Gene name**	**Accession number**	**Primer sequence** ^a^	
NF-kB	XM_342346.4	Sense: 50-AATTGCCCCGGCAT-30	
		Antisense: 30-TCCCGTAACCGCGTA-50	
IRAK1	NM_001127555.1	Sense: 50-GCTGTGGACACCGAT-30	
		Antisense: 30-GCTACACCCATCCACA-50	
TRAF6	NM_001107754.2	Sense: 50-CAGTCCCCTGCACATT-30	
		Antisense: 30-GAGGAGGCATCGCAT-50	
Beta Gusb	NM_017015.2	Sense: 50-GGCTCGGGGCAAATT-30	
		Antisense: 30-GGGGCAGCACGAT- 50	
Gene name	Accession number	Target sequence^b^	Product name
rno-miR-146a	MIMAT0000449	UGAGAACUGAAUUCCAUGGGUU	hsa-miR-146a, LNA PCR primer set, UniRT
rno-miR-191	MIMAT0000440	CAACGGAAUCCCAAAAGCAGCUG	hsa-miR-191, LNA PCR primer set, UniRT

a: Sequences were derived from NCBI (www.ncbi.nlm.nih.gov), b: Sequences were derived from miRBase (www.mirbase.org)

## Results

### Body weight, blood glucose, and plasma insulin

According to our results, induction diabetes in rats for two months showed significant reduction in body weight (218.50±1.91 vs. 468.50±3.70; p<0.01) and fasting blood sugar (504.70±1.87 vs. 110.25±3.30; p<0.01) in the diabetic group compared to that of the control group (p<0.01). In addition, the amount of blood insulin (3.18±0.70 vs. 19.08±2.09; p<0.01) in this group of rats has a significant decrease compared with the control group (p<0.01).

### Expressions of NFKB, TRAF-6, IRAK-1 and miR-146a genes in hippocampus tissue

**Figure 1:**
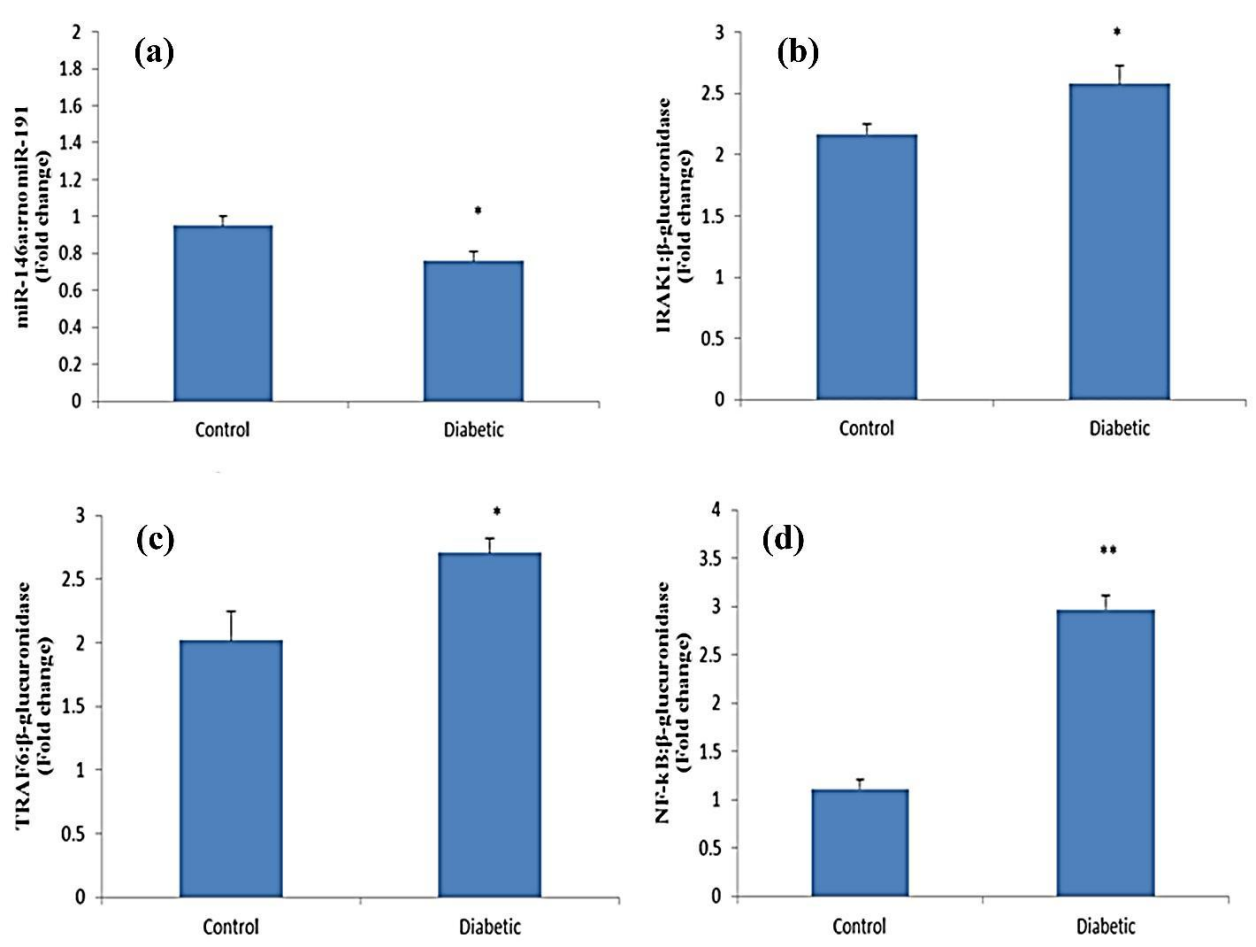
Real-time PCR analysis of miR-146a (a) and mRNA expression level of IRAK1 (b), TRAF6 (c) and NF-kB (d) in the hippocampus of control and diabetic rats at the end of the 2-month uncontrolled diabetes. Bars represent the mean±SD (n=6), *p<0.05 and **p<0.01 vs. the controls

The miR-146a expression level and the IRAK1, TRAF6, and NF-kB mRNA expression levels in the hippocampus are shown in Figure 1. Comparison of the two studied groups indicates a decrease in the amount of expression of miR146a by diabetes in hippocampus tissue (p<0.05). But diabetes causes a significant increase in the expression of TRAF6 and IRAK1 (p<0.05) and NF-kB (p<0.01) genes in the diabetic group compared with the control group.

### Activity of NFKB and apoptosis rate in hippocampus tissue

As presented in Table 2, the amount of NF-KB activity and apoptosis rate has a significant increase in hippocampus tissue in diabetic group compared with the control group (p<0.05).

**Table 2:** ELISA of NF-kB activity and apoptosis rate in hippocampus at the end of the 2-month uncontrolled diabetes.

**Groups**	**NF-kB activity**	**Apoptosis index**
Control	0.392 ± 0.06	0.869 ± 0.09
Diabetic	0.521 ± 0.07*	1.149 ± 0.11*

The values represent mean±SD of 6 animals per group *p<0.05 versus control group.

## Discussion

Results of this study indicate a significant decrease in miR-146a and significant increase in mRNAs of NF-KB, IRAK1, and TRAF6 genes in the hippocampus of diabetic rats as well as increase in NF-KB activity and apoptosis rate in this group compared with the control group. Previous studies have shown that hyperglycemia increases inflammatory mediators like different cytokines, which consequently facilitates inflammation condition and leads to inflammation.^12^ Expression of a large number of inflammation proteins is increased via NF-KB.^20^ In contrast to many studies done about the role of NF-KB in different systems like the immune system, there are a few studies on the role of NF-KB in the nervous system. NF-KB is expressed in neurons and glial cells in central and peripheral nervous systems.^21^ There is not enough information about NF-KB activators in the nervous system; but, it seems that some neurotransmitters like glutamates play activation roles.^21^ NF-KB plays a role in these systems processing via positive regulatory factors by TNF-α, IL-6, and IL-1ß,^22^ which indicates the close relationship between neurological disorders and NF-KB activity in the expression of proinflammatory cytokines.^9^ Therefore, NF-KB can play a role in some processes including synaptic plasticity, synaptic transmission, learning, and memory.^23^ In the present study, increase in the amount of expression of NF-KB gene in diabetic group is significant compared with the control group, which could be a reason for increased apoptosis observed in the hippocampus of the diabetic rats. Therefore, it can be suggested that, like other cells and organs, NF-KB plays a role in the central nervous system in the development of inflammation-associated metabolic diseases like diabetes.^24^ As a regulative factor, miR-146a is induced by Toll-like receptors (TLRs), which depends on NF-KB. Two important adapter molecules, named TRAF6 and IRAK1, participate in the TLR signaling pathway which is known as direct target for miR-146a.^25^ In a study, it has been reported that increase in NF-KB activity reduces TRAF6 and IRAK1 expressions via increasing miR-146a induction, that consequently decreases NF-KB activity (negative feedback regulatory loop).^7^ However, based on the findings in the present study, miR-146 expression is significantly decreased in the hippocampus of diabetic rats, despite the increases observed in NF-KB activity. A similar result is also observed in diabetic wounds where decrease in miR-146a affects NF-KB signaling with a focus on TRAF6 and IRAK1 activities.^26^ Furthermore, a decrease in the amount of miR-146a expression has been reported in a study on endothelial cells of diabetic patients.^27^ However, in a study on sciatic nerve, it has been shown that the miR-146a expression level was increased in the sciatic nerve of diabetic rats compared to their control.^28^ In the present study, it is observed that diabetes decreases miR-146a expression and increases expression of NF-KB, TRAF6, and IRAK1 genes; therefore, it is possible to conclude that negative effects are applied to miR-146a due to the dominance of proinflammatory and inflammatory factors like NF-KB, TRAF6, and IRAK1 in hippocampus tissues, which consequently reduces miR-146a. According to the above discussion, it is possible to report that changes in miR-146a expression and the effects resulted by this change on the signaling pathway of NF-KB can vary depending on tissues and different conditions, which requires further studies about this topic, especially on the nervous system.

## Conclusion

Results of the present study report a decrease in miR-146a expression, increase in expression of TRAF6, IRAK1, and NF-KB genes, and increase in NF-KB activity and apoptosis in the hippocampus of the diabetic rats. Considering the fact that NF-KB is one of the most basic factors of inflammation pathway in most tissues in the body during diabetes, our results imply that the decrease in miR-146a expression increases NF-KB expression and, thus causes the progression of inflammation in the hippocampus. However, future studies are required to provide more explanations about the involved signaling pathways.

## Acknowledgments

The Grant of this study was supported by Neurosciences Research Center, Tabriz University of Medical Sciences, Tabriz, Iran. Our data in this work were derived from the thesis of Ms. Fatemeh Habibi for a Master of Science degree in physiology (thesis serial number: 91/2-2/5).

## Ethical Issues

The study protocol was designed in accordance with NIH guidelines and ethics committee for the use of animals in research at Tabriz University of Medical Sciences (.No: 91/2-2/5/4 Dec 2012)

## Conflict of Interest

The authors have declared that there is no conflict of interest.
